# Nomogram for predicting fulminant necrotizing enterocolitis

**DOI:** 10.1007/s00383-023-05435-9

**Published:** 2023-03-20

**Authors:** Weibo Li, Chen Zhang, Wenli Li, Fanyue Qin, Xiang Gao, Falin Xu

**Affiliations:** 1 Department of Neonatology, The Third Affiliated Hospital of Zhengzhou University, Zhengzhou, Henan China; 2 Branch Center of the Third Affiliated Hospital of Advanced Medical Research Center of Zhengzhou University, Zhengzhou, Henan China; 3 The First Affiliated Hospital of Chongqing Medical University, Chongqing, China

**Keywords:** Fulminant necrotizing enterocolitis, Predictive model, Nomogram, Neonate, Hematological counts

## Abstract

**Background:**

Fulminant necrotizing enterocolitis (FNEC) is the most serious subtype of NEC and has a high mortality rate and a high incidence of sequelae. Onset prediction can help in the establishment of a customized treatment strategy. This study aimed to develop and evaluate a predictive nomogram for FNEC.

**Methods:**

We conducted a retrospective observation to study the clinical data of neonates diagnosed with NEC (Bell stage ≥ IIB). Neonates were divided into the FNEC and NEC groups. A multivariate logistic regression model was used to construct the nomogram model. The performance of the nomogram was assessed using area under the curve, calibration analysis, and decision curve analysis.

**Results:**

A total of 206 neonate cases were included, among which 40 (19.4%) fulfilled the definition of FNEC. The identified predictors were assisted ventilation after NEC onset; shock at NEC onset; feeding volumes before NEC onset; neutrophil counts on the day of NEC onset; and neutrophil, lymphocyte, and monocyte counts on day 1 after NEC onset. The nomogram exhibited good discrimination, with an area under the receiver operating characteristic curve of 0.884 (95% CI 0.825–0.943). The predictive model was well calibrated. Decision curve analysis confirmed the clinical usefulness of this nomogram.

**Conclusion:**

A nomogram with a potentially effective application was developed to facilitate the individualized prediction of FNEC, with the hope of providing further direction for the early diagnosis of FNEC and timing of intervention.

**Supplementary Information:**

The online version contains supplementary material available at 10.1007/s00383-023-05435-9.

## Introduction

Necrotizing enterocolitis (NEC) is a devastating disease that occurs during the neonatal period and is an important contributor to newborn mortality. Approximately 30% of very-low-birth-weight preterm infants die from NEC, but the mortality rates from NEC in extremely low-birth-weight preterm infants are higher, ranging from 30% to 50.9% [1–3]. Surviving infants may develop digestive tract and neurological sequelae.

Fulminant NEC (FNEC) is the most serious NEC subtype, accounting for approximately 10% of NEC cases and causing precipitous decline and death. Additionally, a subset of these infants with FNEC have a particularly virulent form of NEC known as NEC-totalis. Definitions of NEC-totalis vary in the literature but generally refer to patients with massive, near-total bowel ischemia and necrosis [4–7]. The lack of typical imaging findings and abdominal signs makes identification and diagnosis difficult. Counseling parents regarding decision-making in these difficult cases is challenging given the lack of clinical data for these patients. Previous studies have shown that early recognition of the clinical situation, timely diagnosis, and surgical therapy for FNEC may be important in reducing mortality [4]. Although it is known that the pathophysiology of FNEC is shaped by the combined action of feeding, immunological, infectious, drug-induced, and hemodynamic factors [6–9], most studies independently assessed the risk factors predicting FNEC, such as lower lymphocyte counts, lower platelet counts, and full-volume feeding [6, 7]. However, few studies have comprehensively predicted FNEC onset. We hypothesize that the nomogram can predict the occurrence of FNEC. In this study, we synthetically analyzed perinatal risk factors, feeding strategies, clinical characteristics, and hematological counts in infants with FNEC. Importantly, we established a nomogram prediction model with common clinical indicators to determine the predictors of FNEC with the hope of providing further direction for the early diagnosis and timing of intervention.

## Materials and methods

### Study subjects

This study included neonates diagnosed with NEC (Bell stage ≥ IIB) in the newborn intensive care unit (NICU) and Pediatric Surgery Department of the Third Affiliated Hospital of Zhengzhou University between September 2015 and December 2021. Clinical, imaging, and laboratory data of the included patients were retrospectively obtained from the hospital’s medical records. The access and use of this clinical data were approved by the Third Affiliated Hospital of the Zhengzhou University (Project number: 2022-085-01). All participants’ parents provided written informed consent prior to enrollment. Patients were excluded if they had NEC after intestinal surgery, congenital anomalies of the gastrointestinal tract, or inherited metabolic diseases. Patients without complete general demographic records were also excluded. Neonates were stratified into FNEC or NEC groups. FNEC, defined as NEC-totalis or death within 48 h diagnosis of NEC [4–7]. The diagnosis of neonatal shock was based on comprehensive judgment of blood pressure and assessment of regional and global perfusion, such as blood lactate concentrations, skin perfusion, mental status, and urine output [10].

### Clinical information

We collected information regarding maternal factors including pregnancy-induced hypertension, chorioamnionitis, gestational diabetes mellitus, fetal distress, placental abruption, premature membrane, mode of delivery, number of pregnancies, and antenatal steroids. We also collected demographic characteristics and feeding strategies, including gestational age, birth weight, sex, age at first feed, type of nutrition (breast milk or formula), whether full-volume feeds were achieved, and feeding volumes before NEC onset. Full-volume feeds were defined by the nutritionist documented goal feed rate and by neonatologist documentation that the patient was at the goal volume [6]. We recorded NEC features such as the age of onset, primary clinical presentation (abdominal distension, vomiting, fever, bloody stools, and shock), primary radiographic findings (pneumatosis, portal venous gas, pneumoperitoneum), and assisted ventilation at NEC onset.

Finally, we recorded neutrophil, lymphocyte, monocyte, eosinophil, platelet, C-reactive protein, and lactate levels before NEC onset, the day of NEC onset, and day 1 after NEC onset. We also recorded and compared leukocyte counts (including neutrophil, lymphocyte, monocyte, and eosinophil counts) during the first three days after birth.

### Statistical analysis

Statistical analysis was conducted using R software (V.4.1.2, R Foundation for Statistical Computing, Vienna, Austria) and SPSS software (V23.0, IBM, New York, USA), and statistical significance was set at *p* < 0.05. For categorical variables, χ2 or Fisher’s exact test was used. The skewed distribution data were expressed as the median value (interquartile value), and the Mann–Whitney U rank sum test was used to compare the two groups. Logistic regression analysis was performed to identify independent clinical predictors of FNEC [11]. A nomogram was drawn based on the results of multivariate analysis. The discrimination of the model was assessed using the receiver operating characteristic curve [12]. Calibration was assessed using calibration curves, graphic representation of the relationship between the frequency of observations, and probability of prediction, with a 1000-bootstrapped sample of the primary cohort [13]. The clinical usefulness of the model was assessed using decision curve analysis [14].

## Results

### Comparison of general information

A total of 206 neonate cases with confirmed Bell stage ≥ IIB NEC were included in this study (Supplementary figure), of which 40 (19.4%) fulfilled the definition of FNEC. There were 12 neonates who did not have time for surgery because of rapid death, 2 neonates who refused further treatment, such as ventilator support, because of a serious perioperative condition, and 4 neonates in the FNEC group with a poor prognosis, such as short-bowel syndrome.

There was no difference in the maternal information between the FNEC and NEC groups (Table 1).

**Table 1:** Comparison of maternal information

	FNEC (*n* = 40)	NEC (*n* = 166)	*Z/χ2*	*p*
Pregnancy-induced hypertension (%)	12 (30.0)	36 (21.7)	− 1.114	0.265
Chorioamnionitis (%)	3 (7.5)	6 (3.6)	− 0.503	0.615
Gestational diabetes mellitus (%)	8 (20.0)	17 (10.2)	− 1.829	0.067
Fetal distress (%)	7 (17.5)	27 (16.3)	− 0.195	0.845
Placental abruption (%)	9 (22.5)	17 (10.2)	− 1.820	0.067
Premature of membrane (%)	4 (10.0)	41 (24.7)	− 1.948	0.051
Vaginal (%)	12 (30.0)	43 (25.9)	− 0.524	0.600
Multiple pregnancy (%)	12 (30.0)	56 (33.7)	− 0.450	0.653
Antenatal steroids (%)	22 (55.0)	111 (66.9)	− 1.405	0.160

In our entire cohort, the median value (interquartile value) of gestational ages in the FNEC and NEC groups were 31.1 and 31.7 weeks (*p* = 0.181), and birth weights were 1315 and 1545 g, respectively (*p* = 0.124). Regarding feeding strategies, the patients in the FNEC group had less enteral feeding volumes (*p* = 0.006), and fewer patients achieved full-volume feeds, although the difference was not statistically significant (*p* = 0.133). However, there were no significant differences in age at first feed (*p* = 0.649) and type of nutrition (*p* = 0.375) in the other feeding strategy subjects (Table 2).

**Table 2:** Comparison of clinical characteristics and feeding strategies

	FNEC (*n* = 40)	NEC (*n* = 166)	*Z/χ2*	*p*
Gestational age (week)	31.1 (28.6, 33.3)	31.7 (29.1, 34.7)	− 1.337	0.181
Birth weight (g)	1315 (927, 2065)	1545 (1192, 2102)	− 1.538	0.124
Gender—Male (%)	27 (67.5)	99 (59.6)	− 0.914	0.361
First feed day of life (h)	19.5 (10.0, 35.0)	22.0 (12.0, 35.3)	− 0.392	0.649
Breast milk (%)	4 (10.0)	25 (15.1)	− 0.887	0.375
Full feeds achieved (%)	12 (30.0)	71 (42.8)	− 1.503	0.133
Enteral feed volume before onset (ml/kg)	72.5 (12.0, 115.0)	104.0 (63.4, 130.0)	− 2.739	0.006

As summarized in Table 3, after NEC diagnosis, patients in the FNEC group had more severe primary clinical manifestations than those in the NEC group. A higher incidence of shock was observed in the FNEC group (*p* = 0.001). A larger proportion of patients with FNEC received assisted ventilation at NEC onset (*p* < 0.001). Neonates with FNEC were more likely to have pneumatosis, portal venous gas, and pneumoperitoneum on the initial abdominal radiographs, although the difference was not statistically significant. On the day of NEC onset, neonates with FNEC had significantly lower leukocyte (*p* < 0.001) and platelet counts (*p* = 0.009). One day after NEC onset, neonates with FNEC also had lower neutrophil, lymphocyte, monocyte, and platelet counts (*p* < 0.001). Compared with the NEC group, neonates with FNEC had higher lactate levels on the day of NEC onset (*p* = 0.004), and 1 day after NEC onset, neonates with FNEC had significantly higher lactate levels (*p* = 0.000).

**Table 3:** Comparison of radiologic features and laboratory findings

	FNEC	NEC	*Z/χ2*	*p*
	(*n* = 40)	(*n* = 166)		
NEC age onset (d)	16.5 (4.0, 29.8)	13 (7.25)	− 0.096	0.923
Distention at NEC onset (*n*%)	28 (70.0)	93 (56.0)	− 1.608	0.108
Bloody stools at NEC onset (*n*%)	9 (22.5)	59 (35.5)	− 1.571	0.116
Fever at NEC onset (*n*%)	4 (10.0)	5 (3.0)	− 1.936	0.053
Vomit at NEC onset (*n*%)	7 (17.5)	16 (9.6)	− 1.414	0.157
Assisted ventilation at NEC onset intubated (*n*%)	28 (70.0)	45 (27.1)	− 5.079	< 0.001
Shock at NEC onset (*n*%)	15 (37.5)	23 (13.9)	− 3.453	0.001
Pneumatosis at NEC onset (*n*%)	14 (35.0)	34 (20.5)	− 1.945	0.052
Portal venous gas at NEC onset (*n*%)	5 (12.5)	11 (6.6)	− 1.243	0.214
Pneumoperitoneum at NEC onset (*n*%)	3 (7.5)	5 (3.0)	− 1.316	0.188
ANC before NEC onset (10^9^/L)	4.69 (2.34, 6.34)	4.42 (2.69, 6.14)	− 0.022	0.982
ALC before NEC onset (10^9^/L)	2.91 (1.48,4.32)	3.63 (2.52, 4.64)	− 1.879	0.6
AMC before NEC onset (10^9^/L)	0.78 (0.45, 1.66)	0.95 (0.61, 1.28)	− 0.629	0.529
AEC before NEC onset (10^9^/L)	0.37 (0.12, 0.61)	0.43 (0.20, 0.74)	− 1.309	0.191
PLT before NEC onset (10^9^/L)	231 (147, 327)	267 (183, 342)	− 1.172	0.241
CRP before NEC onset (mg/dL)	0.30 (0.20, 0.72)	0.50 (0.21, 1.00)	− 1.573	0.116
Lac before NEC onset (mmol/L)	0.8 (0.5, 1.1)	0.9 (0.6, 1.2)	− 1.365	0.172
ANC at NEC onset (10^9^/L)	2.61 (1.82, 4.80)	4.56 (3.09, 8.26)	− 3.855	< 0.001
ALC at NEC onset (10^9^/L)	1.10 (0.71, 1.94)	2.00 (1.14, 3.66)	− 3.41	0.001
AMC at NEC onset (10^9^/L)	0.29 (0.09, 0.55)	0.7 (0.378, 0.99)	− 4.614	< 0.001
AEC at NEC onset (10^9^/L)	0.05 (0.01, 0.13)	0.13 (0.04, 0.32)	− 3.176	0.001
PLT at NEC onset (10^9^/L)	166 (113, 223)	220 (145, 295)	− 2.615	0.009
CRP at NEC onset (mg/dL)	43.77 (9.10, 82.59)	20.99 (3.44, 67.91)	− 1.925	0.051
Lac at NEC onset (mmol/L)	1.7 (1.2, 3.4)	1.3 (0.9, 1.8)	− 2.887	0.004
ANC day 1 after NEC (10^9^/L)	3.23 (1.97, 5.12)	5.30 (3.31, 8.30)	− 3.99	0.001
ALC day 1 after NEC (10^9^/L)	1.37 (0.82, 2.56)	2.76 (1.72, 4.11)	− 4.249	< 0.001
AMC day 1 after NEC (10^9^/L)	0.40 (0.21, 0.74)	0.94 (0.64, 1.39)	− 5.109	< 0.001
AEC day 1 after NEC (10^9^/L)	0.20 (0.06, 0.61)	0.25 (0.06, 0.61)	− 0.782	0.434
PLT day 1 after NEC (10^9^/L)	108 (56, 226)	199 (123, 259)	− 3.392	0.001
CRP day 1 after NEC (mg/dL)	62.50 (26.57, 106.36)	45.98 (11.53, 87.53)	− 1.799	0.072
Lac day 1 after NEC (mg/dL)	2.4 (1.4, 4.5)	1.5 (1.2, 1.8)	− 3.486	< 0.001

*ANC* absolute neutrophil counts, *ALC* absolute lymphocyte counts, *AMC* absolute monocyte counts, *AEC* absolute eosinophil counts, *PLT* platelet, *Lac* lactate

We also compared leukocyte counts during the first three days after birth between the FNEC and NEC groups. Patients with FNEC had lower leukocyte counts than those with NEC from day 1 until day 3 after birth. However, only neutrophil counts (*p* = 0.002) on day 1 after birth and neutrophil (*p* = 0.000), lymphocyte (*p* = 0.022), and monocyte counts (*p* = 0.006) on day 2 after birth were significantly different (Table 4) among these groups.

**Table 4:** Comparison of leukocyte counts during the first 3 days after birth

	FNEC	NEC	*Z/χ2*	*p*
	(*n* = 40)	(*n* = 166)		
ANC day 1 after birth (10^9^/L)	3.06 (1.74, 5.03)	4.64 (2.73, 6.60)	− 3.33	0.002
ALC day 1 after birth (10^9^/L)	3.69 (2.76, 4.23)	3.69 (2.49, 4.98)	− 0.592	0.554
AMC day 1 after birth (10^9^/L)	0.48 (0.25, 0.99)	0.60 (0.32, 0.96)	− 0.968	0.333
AEC day 1 after birth (10^9^/L)	0.15 (0.07, 0.38)	0.19 (0.09, 0.35)	− 1.345	0.179
ANC day 2 after birth (10^9^/L)	4.40 (3.06, 5.96)	6.73 (4.24, 9.59)	− 3.586	0
ALC day 2 after birth (10^9^/L)	2.11 (1.29, 3.09)	2.55 (1.77, 3.85)	− 2.286	0.022
AMC day 2 after birth (10^9^/L)	0.53 (0.28, 0.73)	0.74 (0.46,1.16)	− 2.739	0.006
AEC day 2 after birth (10^9^/L)	0.09 (0.28, 0.24)	0.10 (0.04, 0.23)	− 0.897	0.37
ANC day 3 after birth (10^9^/L)	2.58 (1.65, 6.22)	4.31 (2.38, 7.21)	− 2.11	0.035
ALC day 3 after birth (10^9^/L)	2.24 (1.31, 3.73)	4.31 (2.38, 7.22)	− 1.934	0.053
AMC day 3 after birth (10^9^/L)	0.68 (0.42, 1.17)	4.31 (2.38, 7.21)	− 1.378	0.168
AEC day 3 after birth (10^9^/L)	0.11 (0.04, 0.26)	0.23 (0.10, 0.27)	− 1.122	0.262

*ANC* absolute neutrophil counts, *ALC* absolute lymphocyte counts, *AMC* absolute monocyte counts, *AEC* absolute eosinophil counts

### Multivariate regression analysis of the occurrence of FNEC

The above possible influencing factors were used as independent variables, and whether FNEC occurred was used as a dependent variable in the multivariate analysis. The analysis results showed that assisted ventilation after NEC onset; shock at the time of onset; feeding volumes before onset; neutrophil counts on the day of NEC onset; and neutrophil, lymphocyte, and monocyte counts on day 1 after NEC were independent factors influencing the occurrence of FNEC (Table 5).

**Table 5:** Results of the multivariate logistic regression analysis

Factors	*B*	S. E	Wald	*p*	OR	95% CI
Ventilation	1.494	0.4907	3.04	0.002	4.456	1.703–11.658
Shock	1.100	0.543	2.03	0.043	3.003	1.037–8.699
Volume Onset	− 0.013	0.005	− 2.74	0.006	0.393	0.202–0.767
ANConset	− 0.146	0.068	− 2.14	0.032	0.500	0.245–0.939
ANCday1	− 0.17	0.086	− 1.98	0.048	0.434	0.190–0.993
ALCday1	− 0.293	0.126	− 2.01	0.045	0.475	0.230–0.982
AMCday1	− 1.561	0.543	− 2.87	0.004	0.287	0.123–0.673
Constant	− 2.148	0.791	2.71	0.007		

*Ventilation* assisted ventilation after NEC onset, *shock* shock at NEC onset, *VolumeOnset* feeding volumes before onset, *ANConset* neutrophil counts on the day of FNEC onset, *ANCday1* neutrophil counts on the day 1 after NEC, *ALCday1* lymphocyte counts on the day 1 after NEC, *AMCday1* monocyte counts on the day 1 after NEC, *OR* odds ratio, *CI* confidence interval

### Nomogram development and nomogram validation

#### Nomogram model for predicting the risk of FNEC

**Fig. 1:**
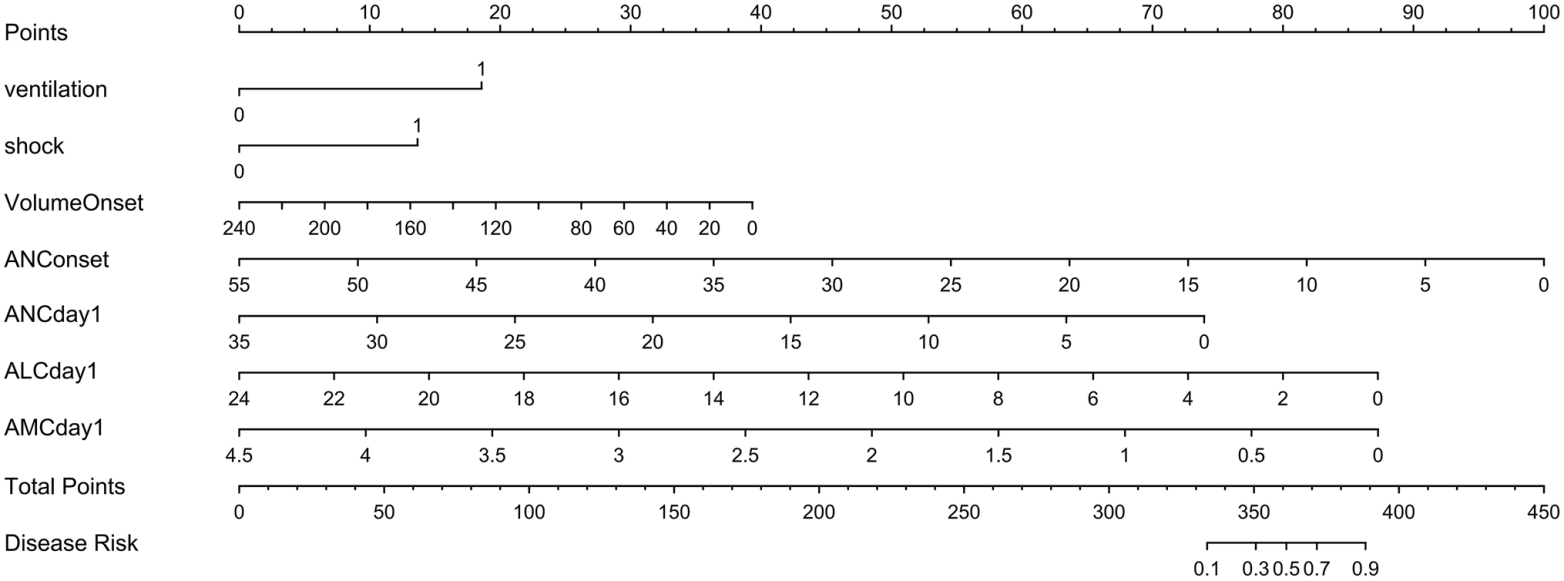
A nomogram was used to determine the probability of FNEC severity. *Ventilation* assisted ventilation after NEC onset, *shock* shock at the time of onset, *VolumOnset* feeding volumes before onset, *ANConset* neutrophil counts on the day of FNEC onset, *ANCday1* neutrophil counts on day 1 after NEC, *ALCday1* lymphocyte counts on day 1 after NEC, *AMCday1* monocyte counts on day 1 after NEC

A nomogram for severity was designed and assimilated using the predictors. Predictor points were found on the uppermost point scale that matched each patient variable and were added. The total points extrapolated to the bottom scale show the percentage probability of the severity. (Fig. 1).

#### Nomogram validation

**Fig. 2:**
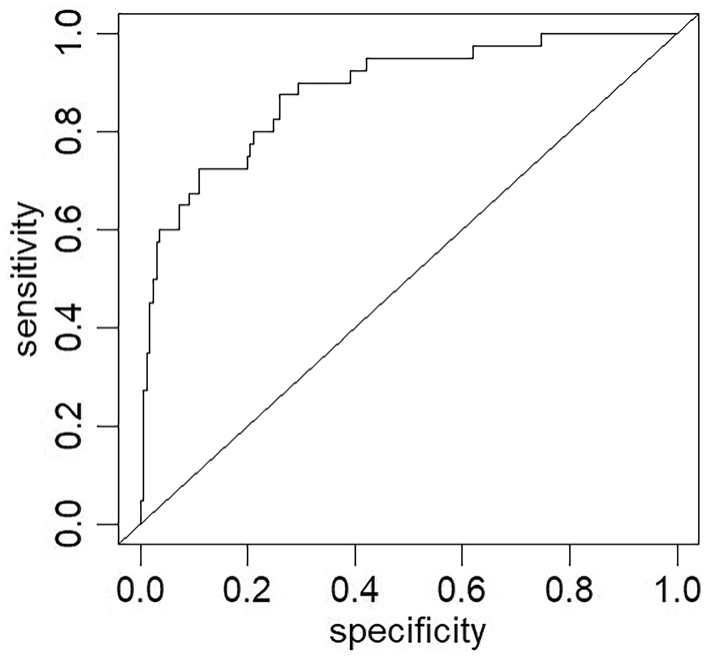
Nomogram validation was performed using receiver operating characteristic curves in the main cohort. *ROC* receiver operating characteristic, *AUC* area under the ROC curve

The nomogram exhibited excellent power of discrimination, with an area under the curve of 0.884 (95% confidence interval: 0.825–0.943) in the main cohort. (Fig. 2).

**Fig. 3:**
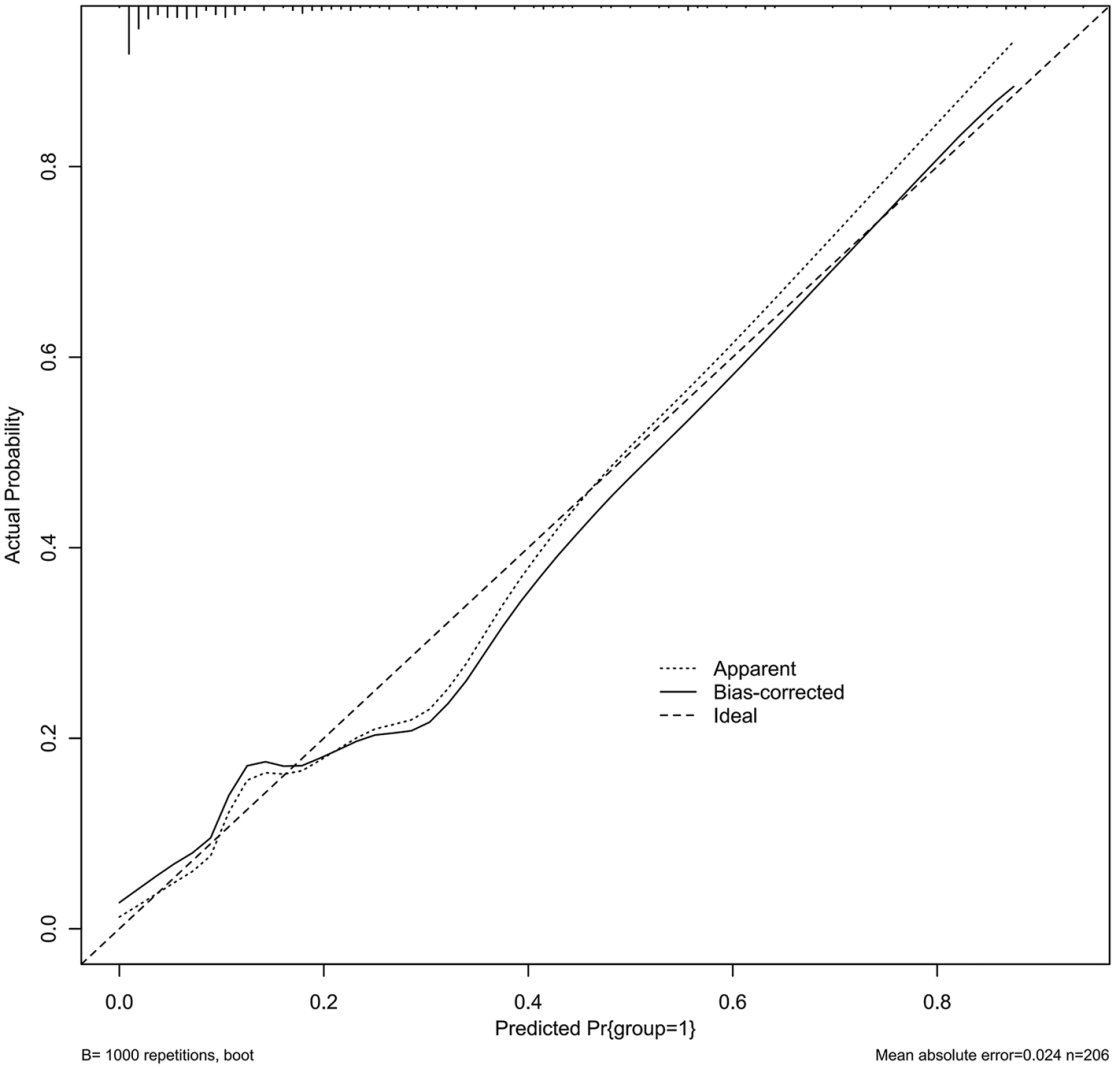
A nomogram was used to determine the probability of FNEC severity

In the calibration curve analysis of the main cohort, the dotted line represents the entire cohort (*n* = 206), and the solid line depicts the results after bias correction by bootstrapping (1000 repetitions). (Fig. 3).

### Clinical use

**Fig. 4:**
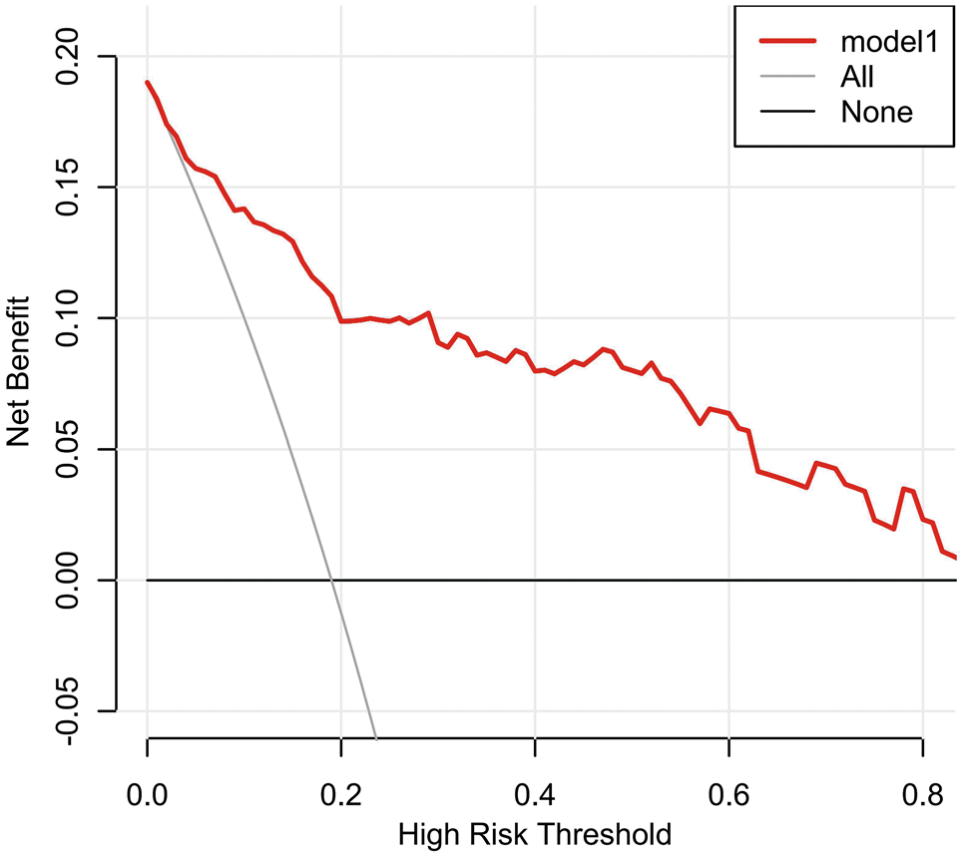
A nomogram was used to determine the probability of FNEC severity

The y-axis indicates the net benefit. The nomogram is represented by a red line. The gray line indicates the presumption that all neonates had FNEC. The horizontal line indicates the presumption that no patients had FNEC. The decision curve analysis curve is at the top right of the two curves and shows the obvious net benefits of the predictive nomogram. (Fig. 4).

## Discussion

Previous studies have determined that the main risk factors for FNEC are hematological abnormalities and rapid feeding escalation using multivariate regression analysis. Our findings identified seven factors related to the risk of FNEC through single factor and multivariate regression analyses, including assisted ventilation after onset; shock at onset; feeding volume before onset; neutrophil counts on the day of onset; and neutrophil, lymphocyte, and monocyte counts on day 1 after NEC onset, all of which were used to establish a nomogram for predicting FNEC. In recent years, prognosis models based on statistical methods have rapidly developed; however, to the best of our knowledge, this is the first nomogram study to predict FNEC. Routine blood examination is easily analyzed, often available around the clock in all kinds of medical institutions. To know that a larger decrease in blood counts corresponds to increased odds of severe NEC at NEC onset can be helpful in the clinical management of NEC infants. Based on the area under the curve and calibration curve evaluation, this prognostic model showed good discriminatory ability, calibration, and clinical usefulness.

Garg et al. reported that a fulminant disease course was associated with a more extensive clinical disease with prominent radiological signs and a greater need for assisted ventilation and inotropes before and after surgery [6]. Lin et al. reported that FNEC is characterized by urgent onset and prompt deterioration [15]. In the current study, we found that assisted ventilation and shock at FNEC onset were predictors of FNEC. However, these characteristics also exist at the onset of neonatal sepsis. Therefore, although both are predictors of FNEC in our nomogram model, they still need to be combined with other factors to predict the onset of FNEC. In previous studies, higher lactate levels were associated with NEC, and statistically higher blood lactate levels were found in non-survivors of NEC compared to those of survivors [16, 17]. Lactate level was found to be a valuable prognostic factor for NEC in preterm neonates with NEC [18]. However, in our study, lactate level could not predict FNEC, which may be related to the treatment of tissue perfusion, such as rectification of acid intoxication, use of vascular active medicine, and increased blood capacity.

In Garg’s predictive model, for a 5% increase in neutrophils on the day of NEC diagnosis relative to pre-NEC neutrophil percentages, the odds of FNEC decreased by 16% [6]. Lambert et al. reported lower lymphocyte counts in neonates with FNEC [19]. However, they did not reveal other relationships with blood values. Our findings suggest that neutrophil counts at NEC onset and neutrophil, lymphocyte, and monocyte counts at 24 h after NEC onset were both predictors of FNEC, which suggests the need to monitor blood count changes over time. Systemic inflammation during NEC has been associated with altered platelets and leukocytes [20]. At the onset of NEC, neutrophils reach the intestinal lamina propria as first responders to sites of inflammation to perform a variety of antimicrobial functions, such as phagocytosis and formation of massive amounts of reactive oxygen species and other toxic molecules, which not only effectively destroy pathogens but also cause mucosal injury to a certain extent [21]. However, in the course of severe NEC, numerous neutrophils in the peripheral blood are rapidly recruited to the intestine and peritoneum or attached to the wall of small blood vessels, resulting in a large reduction of neutrophils in the peripheral blood circulation. As part of the normal gut inflammatory response, neutrophils are recruited to sites of infection or inflammatory stimuli within minutes, and the response peaks at 24–48 h [22], which is similar to the change in neutrophil counts after FNEC onset in our data. Previous studies have provided evidence that NEC can be considered as a lymphocyte-mediated disease. NEC development requires an influx of lymphocytes into the lamina propria intestine via toll-like receptor 4 signaling in the intestinal epithelium [23]. Monocyte-derived intestinal macrophages participate in the gut wall infiltration classically observed in NEC, and the rapid efflux of monocytes to NEC lesions is likely to deplete the limited circulating pool of monocytes in premature neonates [24]. This may explain why blood count changes can predict FNEC. However, the mechanism underlying NEC-related thrombocytopenia remains unclear. This mechanism may be linked to platelet activation and the consumption of platelets in microthrombi formed in the intestinal microvasculature during NEC-like injury [25, 26]. Thrombocytopenia is a common clinical finding in NEC, typically observed within 24–72 h after NEC development [27]. This may explain why the platelet count was not a sensitive predictor.

In our nomogram model, enteral feed volume was a predictor of the occurrence of FNEC. Hartman et al. reported that full-volume feeding was associated with FNEC on multivariate analysis [7], and Lambert et al. identified that more cases in the fulminant group had a faster escalation of feeding preceding the development of NEC [16]. This suggests that feeding practice affects the development of this more virulent form of NEC.

Our study also found lower neutrophil counts in neonates with FNEC on the first day after birth, and neutrophil, lymphocyte, and monocyte counts were still lower in the FNEC group on the second day after birth compared with those in the NEC group. In addition, the leukocyte counts in the FNEC group were lower than the average values reported in the existing literature [28–30]. Tröger et al. reported that the numbers of white blood cells and neutrophils were diminished in small for gestational age infants at birth, and on day 3, these preterm infants had a higher risk for combined adverse outcomes [31]. Nguyen et al. reported that preterm pigs had very low blood neutrophil and lymphocyte counts and commonly showed poor weight gain and diarrhea [32, 33]. This suggests that there may be a connection between intestinal diseases and systemic immunity; in other words, neonates with immature systemic immune development who also have an immature digestive system may have an increased risk of gastrointestinal diseases, such as NEC. Therefore, individual feeding and therapy are necessary for premature babies with lower leukocyte counts. This interesting clinical phenomenon may provide clues for identifying high-risk infants with severe NEC; however, the causes need to be further explored.

This study has a few limitations. First, it was conducted retrospectively in a single institution. The nomogram must be confirmed in multicenter studies with large sample sizes. Second, this was a retrospective study based on a review of medical records, and patients with incomplete medical records were excluded. Third, the mechanisms of hematological counts and FNEC need to be further explored.

## Conclusions

We developed a nomogram with seven factors, including assisted ventilation after NEC onset; shock at NEC onset; feeding volume before NEC onset; neutrophil counts on the day of NEC onset; and neutrophil, lymphocyte, and monocyte counts on day 1 after NEC onset. This nomogram may help in the individualized prediction and treatment of FNEC.

### Supplementary Information

Below is the link to the electronic supplementary material.

Supplementary file1 (DOCX 25 KB)

## Data Availability

The raw data supporting the conclusions of this article will be made available by the authors, without undue reservation.

## Abbreviations

FNEC: Fulminant necrotizing enterocolitis
NEC: Necrotizing enterocolitis
NICU: Newborn intensive care unit
ANConset: Neutrophil counts on the day of FNEC onset
ANCday1: Neutrophil counts on day 1 after NEC
ALCday1: Lymphocyte counts on day 1 after NEC
AMCday1: Monocyte counts on day 1 after NEC
ROC: Receiver operating characteristic
AUC: Area under the ROC curve
