# Supplementary material for: Nomogram for predicting fulminant necrotizing enterocolitis
Source: Pediatr Surg Int. 2023 Mar 20;39(1):154. doi: 10.1007/s00383-023-05435-9 (PMC10027821; doi:10.1007/s00383-023-05435-9)
Supplement: Supplementary file 1 — Supplementary file1 (DOCX 25 KB) [file 383_2023_5435_MOESM1_ESM.docx]

Neonates with NEC diagnosis n=226

Patients with NEC (≥IIB) diagnosis during 2015.9-2021.12 n=232

Total verified NEC (≥IIB) included n=206

Excluded

age＞28d n=6

Excluded

NEC after intestinal surgery n=2

congenital malformation n=5

inherited metabolic diseases n=6

uncomplete general demographic records n=7

Supplemental material 1 Flow diagram of patient selection
